# Beyond misconduct: forging an ethical future in academia

**DOI:** 10.1007/s12630-025-03021-2

**Published:** 2025-07-14

**Authors:** Britta S. von Ungern-Sternberg, Karin Becke-Jakob

**Affiliations:** 1 Department of Anaesthesia and Pain Medicine, Perth Children’s Hospital, 15 Hospital Avenue, Nedlands, WA 6009 Australia; 2 Perioperative Medicine Team, The Kids Research Institute Australia, Nedlands, WA Australia; 3 Institute for Paediatric Perioperative Excellence, The University of Western Australia, Perth, WA Australia; 4 Division of Emergency Medicine, Anaesthesia and Pain Medicine, The University of Western Australia, Perth, WA Australia; 5 Department of Anaesthesia, University Children’s Hospital Basel, Basel, Switzerland

**Keywords:** author, authorship, authorship abuse, editor, leadership, misconduct, publishing, speaking up, whistleblower

Authorship not only acknowledges significant intellectual contributions to research and publications but also acts as a form of academic currency, a surrogate marker of scholarly productivity. Authorship also plays a key role in shaping a researcher’s professional standing and career progression.^1^ Nevertheless, pervasive issues of authorship misconduct threaten the integrity of academic publishing. In this Reflections article, we examine these challenges, contextualize the issue within broader systemic misconduct and toxic leadership, and propose meaningful reforms. Without decisive action, these unethical practices will continue to undermine trust in research and stifle innovation by marginalizing deserving contributors.

We recently published our experience with editorial misconduct^1^ that ensued after being invited to contribute a review article^2^ to a special edition of a different, established, nonpredatory journal—to increase awareness and encourage others to also speak up. This editorial was followed by our recent reflections on toxic leadership as an enabler.^3^ To fully orient readers unfamiliar with our previous work, we summarize the key incidents that underpin this Reflections article. Following our publication on editorial authorship misconduct,^1^ we received numerous responses from colleagues across diverse institutions, underscoring the widespread and systematic nature of misconduct and echoing concerns about power imbalances, gendered discrimination, and institutional complicity. Correspondents reported similar concerns in disciplines ranging from the biomedical sciences to the humanities, highlighting the universal nature of the problem. Institutional inertia and fear of retaliation often discourage whistleblowers, exacerbating the problem and allowing unethical practices to persist.

In parallel, our second article explored the role of toxic leadership in academia, particularly how leadership styles rooted in coercion, favouritism, and intimidation can create environments ripe for misconduct.^3^ Toxic leaders often control access to research opportunities, funding, and authorship credits and may use these as tools for manipulation or exclusion. This power imbalance reinforces a culture of silence, where early-career researchers feel pressured to conform or face retaliation. Authorship misconduct and toxic leadership are not entirely separate phenomena; they can be mutually reinforcing, and both must be addressed to achieve meaningful change.

We are often asked who the players are, but viewed through a "big picture" lens, it does not matter—we want to highlight the general problem, promote cultural change, and propose actionable solutions for fostering integrity in academia. We solely report on "the tip of the iceberg"; many more experiences have been left untold. Let us not look back but forwards at the "whole iceberg," at our overall academic culture—and learn from it.

In response to our editorial,^1^ colleagues voiced both astonishment and admiration that we broke the unspoken code and dared to speak up and disrupt the spiderweb of connections of influential people, disregarding the well-meant warnings about the potential negative repercussions it could have on us, our families, and our careers. Nevertheless, it seems to have encouraged others to voice their concerns. We continue to be inundated with messages (e.g., in person and via phone, text, social media, and e-mail) from colleagues worldwide, sharing their personal experiences and expressing support. Unfortunately, most people reported staying silent and accepting the misconduct, as this was simply the easier, and even the safer, choice, inevitably further empowering the perpetrators. Very few reported to have spoken up. This groundswell of feedback highlighted several key issues that underscore the pervasive nature of authorship misconduct and its impact on individuals and the academic community.

## Observations and patterns

Strictly preserving confidentiality, we synthesize herein only key reflections from our personal perspective.

### Widespread nature of misconduct

Reports came from all over the globe and diverse fields, underscoring the universality of the issue. Most respondents revealed their reluctance to speak up, citing fear of retaliation or harm to their careers. This silence perpetuates a cycle where perpetrators remain unchallenged, and misconduct becomes normalized.

### Gendered dynamics

The majority of those who contacted us were not people who identified as men, highlighting a potential gendered dimension to vulnerability in academia. Nevertheless, we acknowledge that, since the senior authors of the index article^2^ are women, the reporting might have been biased. While men also reported experiences of coercion, the systemic imbalances appeared to disproportionately affect people other than men, especially in fields dominated by men. Nevertheless, this should not exclude how gender bias intersects with race, socioeconomic status, age/stage of career, and other social identity markers.^4,5^

### Power structures and influence

What we described as the “normalization of the misconduct” and the overpowering theme of “helplessness”^1^ was also reflected in the responses. While some reported editorial misconduct, pressure from within institutions, societies, divisions, and departments was a more prominent theme, often linked with concrete threats and punishing behaviour if the requests for authorship or cutting corners were not granted. Sadly, the few who reported having spoken up all shared stories of either not being believed or of severe repercussions and threats, including negative consequences not just for themselves but also for their teams and sometimes even their families.

Many of the alleged perpetrators featured in the reports are senior academics with wide-reaching influence in societies, institutions, departments, and editorial boards, forming protective networks that make it hard for junior colleagues to challenge unethical behaviour. Alarmingly, some alleged perpetrators appeared repeatedly in independent reports from various institutions and countries. Indeed, there are always two sides to every story, but a common pattern emerges. It was notable that these individuals often struggle to retain early- and mid-career researchers in their teams long term, often driving them away and marginalizing them as they become more independent researchers to dampen their upward trajectory—unlike truly inspirational mentors who support growth, even beyond their own expertise.

Power imbalances in the academic world; exclusive, tight-knit circles of mutual promotion and loyalty among senior academics; and fear of retaliation or career damage create significant barriers to speaking up. These barriers are further reinforced by a lack of psychological safety, an innate fear of repercussion, a fear of losing their standing, and a fear that everything they worked so hard for would fall to pieces.

## Framework for proposed solutions

To address these systemic issues, we propose that the following measures^6,7^ be considered by the academic community and leadership:

### Creation of safe reporting mechanisms

- Establishment of anonymous pathways for reporting academic misconduct, possibly mediated by journals or professional societies.
- Appointment of institutional integrity advisors to guide individuals facing ethical dilemmas.
- Creation of peer-to-peer support welfare officer positions in departments/divisions to support staff and reduce bullying, undue pressures, and threats. If already present, implementation of processes to ensure that these are truly independent and work within a safe environment. It is important to note that we refer in this context to repeated, intentional behaviour by one or more individuals who intend to harm, intimidate, or exert power over another individual. Undue pressure and threats can be expressed in various ways, including verbal, written, and physical means, and can be direct or indirect.
- Increasing support for mentoring programs within and outside institutions to help researchers build larger networks for collaboration, support, and advice during challenging situations.

### Promotion of transparency and accountability

- Implementation and execution by journals and academic institutions of clear policies on authorship criteria and conflicts of interest.
- Revisiting the issue of an arbitrarily set maximum number of byline authors or affiliations for large collaborative, interdisciplinary, and interinstitutional work to acknowledge the often substantial input from many collaborators and reflect how many researchers are actively involved in several institutions. This artificial restriction is counterintuitive to the urgent need for equity, fairness, and transparency. While authorship numbers should not be artificially inflated, they should also not be artificially constricted. Otherwise, this may not only lead to the lack of recognition of people’s work and a possible reduction in perceived accountability by non-byline authors, but inevitably, it will also fuel the “club-like” structures and networks to put “their own” into the byline while banishing others to the large collaborator lists. This is likely to put even larger pressure on early- and mid-career investigators to “comply and play the game.” These “club-like” structures are networks where people build relationships and exert influence, potentially excluding or marginalizing other groups of people (e.g., “boys’ clubs” marginalizing women, nepotism, or cronyism favouring relatives or friends).
- Establishment of regular audits of editorial and institutional practices to help identify patterns of abuse.

### Fostering a culture of integrity

- Conduct of regular sessions at local, national, and international conferences on publication ethics and misconduct to increase knowledge and awareness.
- Diversification of editorial board memberships to increase equitable, diverse, and inclusive representation; to ensure that different perspectives, voices, and experiences are represented; and to reduce bias, stereotypes, and gaps in content coverage. Diverse editorial boards foster a culture of inclusivity, equity, transparency, and fairness and will increase the trust the readership will put into the publications.
- Encouragement of an open dialogue about academic misconduct and fostering of environments where individuals feel safe speaking up.
- Establishment of structures where senior academics model ethical behaviour, mentor inclusively, and support diverse representation in their teams, departments, institutions, societies, and/or editorial boards.

## Call to action

We urge all members of the academic community to reflect on their practices and ask critical questions. Are our decisions transparent and fair? Do we challenge unethical behaviour or unintentionally normalize it through silence? Do we support the growth of mentees and collaborators from diverse backgrounds, fostering environments where they choose to stay and thrive?

Do we promote people on the basis of merit or on the basis of familiarity and convenience? Are our authorship practices clear and equitable? Do we recognize and confront our own biases in how we assign opportunities, leadership, and visibility?

Above all, do we act when we witness misconduct? Do we offer real support to those who speak up, even when it carries personal risk? Are our decisions in journals, societies, and conferences inclusive and transparent, or are they driven by personal networks? The Table summarizes some reflections on academic practice.

**Table:** Reflections on academic practice

Overarching theme	Questions	Examples
Fairness and transparency in decision-making^8^	• Do I and those around me make decisions fairly and transparently? • How do I choose the people I work with or who gets to speak or lead? • Are my decisions team-based or individual?	• Deciding project leads or conference speakers openly and collaboratively
Ethics and integrity^9^	• Do I challenge unethical behavior, or do I normalize it through silence? • Do I act if I see a problem? • Do I withdraw a manuscript when I know of unethical behavior?	• Speaking out against misconduct, even at personal cost • Withdrawing a paper owing to integrity issues
Mentorship and collaboration	• Do I have mentees? • How do I treat them? • Do collaborators choose to work with me over time? • Do I give them space to grow?	• Supporting mentees to build independent track records^10–12^ • Long-term collaboration based on mutual respect and growth
Motivations and culture	• What motivates me: personal success or team success? • Is there a sense of collective joy when projects end? • How is authorship decided?	• Choosing to prioritize team recognition over personal acclaim • Transparent authorship discussions
Bias and inclusion	• What are my biases and preconceptions? • Am I doing enough to reduce bias? • Do I look for diversity in teams and decisions?	• Ensuring diverse representation when forming panels or teams^13,14^ • Reflecting on implicit bias during selection processes
Openness and listening^15^	• Do I lend an open ear? • Do I listen to all sides? • Am I open to being challenged by junior staff?	• Creating spaces for feedback and challenge regardless of seniority
Responsibility and courage	• Do I act if I see something wrong? • Do I support whistleblowers publicly or only in private?	• Standing up for others, even at a personal cost • Avoiding complicity through inaction, as the standard you walk past is the standard you accept
Community and gatekeeping	• How do we select speakers, reviewers, or editorial board members? • Do we favour our own network? • Are we seeking the most knowledgeable people, those with good presentation skills, or just familiar ones?	• Prioritizing expertise and diversity over convenience or favoritism

True reform starts with critical self-inquiry. Before we point fingers, let us strive to be inclusive, ethical, and generous colleagues and mentors—those who lead with principle, not convenience.

If we all do that, not only will there be better behaviour overall, but there will also be a better culture; there will be more safety and peer-to-peer control. That is when we can collectively address the cracks in our academic system; that is when academic integrity will blossom again, and we can all focus our energy on our actual tasks—to perform and promote good science to improve the outcomes of our patients, rather than police bad behaviour. We hope the academic climate will change and the iceberg of misconduct will melt, disappear, and become part of the ocean again, transforming our academic culture into one of transparency, equity, fairness, and mutual respect.
